# Effects on Sleep Quality and Bioelectrical Parameters of Cellular Health After Administration of a Plant Protein Supplement Combined with Ashwagandha and Rhodiola: A Randomized Controlled Trial

**DOI:** 10.1007/s11130-026-01585-y

**Published:** 2026-11-11

**Authors:** A Molina-López, A Leo-Romero, M Molina-López, S Polo-Marco, D Domínguez-Balmaseda, G García-Pérez-de-Sevilla, E Casanova, H Moya-Amaya, D Granizo- Bermejo, F.J. Berral-de la Rosa

**Affiliations:** 1 Udinese Calcio Nutrition Deparment, Udine, Italy; 2 Centre for Research into Physical and Sporting Performance (CIRFD), Universidad Pablo de Olavide, Seville, Spain; 3 Molina Pharmacy, Pozoblanco, Cordoba Spain; 4 Marta Marco Pharmacy, Yecla, Murcia Spain; 5 Faculty of Medicine, Health and Sports, Department of Real Madrid Graduate School, Universidad Europea de Madrid, Villaviciosa de Odón, Spain; 6 Unifarco SPA, Santa Giustina, BL Italy

**Keywords:** Withania somnifera, Rhodiola rosea, Adaptogens, Phase angle, Bioimpedance, Sleep disorders

## Abstract

Sleep disturbances affect a significant portion of the global population, impacting health and quality of life. While plant-based proteins and adaptogens such as ashwagandha and rhodiola have shown individual benefits, their combined effects remain poorly understood. This study evaluated whether a nutritional intervention combining plant protein with ashwagandha-rhodiola could improve sleep quality in middle-aged adults. A randomized, double-blind, placebo-controlled trial was conducted with 100 participants aged 35-70 years, assigned to VERUM (*n*=50) or PLACEBO (*n*=50) groups. The VERUM group received plant protein (27 g/day: pea, rice, hemp isolate) plus adaptogen tablets (Rhodiola rosea 300 mg + Withania somnifera KSM-66® 500 mg) for 30 days. Sleep quality was assessed using the validated Sleep Quality Questionnaire (SQQ) completed daily. Phase angle was measured by bioimpedance (BIA-TELELAB) pre- and post-intervention. Data were analyzed using longitudinal mixed models and non-parametric tests. Seventy-seven participants completed bioimpedance analysis (VERUM group; n= 38, PLACEBO group n=39; mean age 47.8±8.2 years), and sixty-one completed sleep assessments (VERUM group n=32; PLACEBO group n=29). The VERUM group showed highly significant improvements: sleep difficulty reduced by 56% (*p*<0.001, d=0.91), daytime sleepiness decreased by 60% (p<0.001, d=0.85), and total sleep score improved by 58% (*p*<0.001, d=0.96), representing a 3.7-fold greater improvement than placebo. Phase angle changes were minimal in both groups. No adverse events were reported. The combination of plant protein with ashwagandha-rhodiola for 30 days produces substantial, clinically relevant improvements in sleep quality with an excellent safety profile, with statistically greater improvements compared to placebo.

**Supplementary Information:**

The online version contains supplementary material available at https://doi.org/10.1007/s11130-026-01585-y.

## Introduction

Quality sleep is essential for maintaining physical and mental health, yet paradoxically we live in an age where sleep disorders have become increasingly prevalent. Current estimates suggest that between 30% and 40% of the adult population experiences some type of sleep-related problem [1, 2]. These disorders are associated with serious metabolic consequences, including insulin resistance, weight gain, and increased cardiovascular risk [3, 4].

Traditionally, the pharmacological management of insomnia has relied on benzodiazepines and hypnotics, but these medications have significant limitations: they can be addictive, produce undesirable side effects and, in some cases, worsen sleep architecture in the long term [5]. This reality has led to growing interest in natural alternatives.

Among emerging nutritional strategies, protein supplementation has attracted attention for its potential to modulate sleep through various mechanisms. Proteins are a source of tryptophan, a precursor of serotonin and melatonin, key neurotransmitters in the regulation of the sleep-wake cycle [6]. In addition, protein intake can influence nocturnal glycaemic stability and the synthesis of bioactive peptides with sedative properties [7, 8]. However, studies on protein and sleep have shown mixed results, possibly because the effects depend on the timing of administration, dosage, and amino acid composition [9].

At the same time, adaptogens—medicinal plants that help the body adapt to stress—have shown promising effects on sleep. Ashwagandha (*Withania somnifera*) is perhaps the most studied adaptogen in this context. Several clinical trials have documented that ashwagandha improves sleep quality, reduces sleep latency, and decreases night-time awakenings, probably through its modulatory action on the hypothalamic-pituitary-adrenal (HPA) axis and its gamma-aminobutyric acidergic (GABAergic) effects [10–12]. Rhodiola (*Rhodiola rosea*), another well-characterised adaptogen, has shown benefits in reducing fatigue and improving daytime alertness, although its direct effect on sleep has been less explored [13, 14].

What is particularly intriguing is the possibility that these components—proteins and adaptogens—may act synergistically. Beyond sleep, there is growing interest in understanding how nutritional interventions affect cellular health. Phase angle, measured by bioelectrical impedance analysis (BIA), has emerged as a promising marker of cellular integrity and nutritional status [15, 16]. This parameter reflects the ability of cell membranes to store electrical energy and has been associated with clinical prognosis in various conditions, from chronic diseases to ageing [17]. Although some studies have suggested that protein supplementation could improve phase angle by increasing body cell mass [18], evidence in healthy adults is limited.

In this context, we designed the TECOS (targeted enhancement of cellular and roganismal sleep) study with a dual objective: first, to evaluate whether the combination of plant protein with ashwagandha and rhodiola could improve sleep quality beyond the placebo effect; second, to explore whether this intervention had measurable effects on cellular health, as assessed by phase angle.

## Materials and Methods

### Study Design

The TECOS study is a 30-day, randomised, double-blind, placebo-controlled, parallel-group clinical trial conducted at three sites in Spain: Seville, Yecla (Murcia), and Pozoblanco (Córdoba). The protocol followed CONSORT guidelines for parallel-group trials and was prospectively registered (NCT07461402). All procedures were approved by the relevant Research Ethics Committee (C.P. PIS-CC C.I. 2061-N-21) and conducted according to the Declaration of Helsinki and Good Clinical Practice. Written informed consent was obtained from all participants.

### Participants

Adults aged 35–70 years with subjective complaints of poor sleep quality were recruited at the three study sites. Detailed inclusion and exclusion criteria are provided in Supplementary Methods S1.

### Sample Size

Sample size was calculated using G*Power software (version 3.1.9.7; Heinrich Heine University, Düsseldorf, Germany) for the comparison of two independent means (two-tailed). The calculation assumed a significance level (α) of 0.05, a statistical power of 80% (1 − β = 0.80), an expected between-group difference of 2 points in the total Sleep Quality Questionnaire (SQQ) [19] score, and a common standard deviation of 3 points, corresponding to a standardized effect size (Cohen’s *d*) of 0.67. The expected difference was considered clinically meaningful, whereas the assumed variability was based on the psychometric characteristics reported for the SQQ in validation studies. Under these assumptions, 36 participants per group were required. To compensate for an anticipated dropout rate of approximately 25%, and to ensure balanced allocation between groups, the recruitment target was conservatively increased to 50 participants per group (100 participants in total).

### Randomisation and Blinding

Participants were randomised 1:1 to VERUM or PLACEBO using a computer-generated sequence with permuted blocks of variable size (4–6 participants) to ensure group balance during recruitment.

The study was double-blind: participants, care providers, outcome assessors, and data analysts remained blinded to group allocation throughout the study. Blinding was maintained at the level of product manufacturing and packaging by an external certified manufacturer not involved in trial design, data collection, or analysis. VERUM and PLACEBO products were indistinguishable in appearance, taste, texture, and packaging, and were labelled only with non-informative alphanumeric codes. Further details on sequence generation, allocation concealment, and code management are provided in Supplementary Methods S2.

### Interventions

VERUM group: Participants received a plant protein powder supplement (27 g/day) containing pea, rice and hemp protein isolate. The protein blend was manufactured under food-grade quality standards, and its amino acid profile (including tryptophan content and essential amino acid distribution) was provided by the manufacturer based on batch-specific compositional analysis. In addition, participants received two daily adaptogen tablets with Rhodiola rosea and Withania somnifera (KSM-66^®^).

PLACEBO group: Participants received an isocaloric placebo powder (maltodextrin with flavouring identical to the active product) and placebo tablets identical in appearance, taste, and texture to those of the VERUM group, with the same administration instructions. Detailed botanical extract characterization and quality-control procedures are provided in Supplementary Methods S3.

### Compliance and Adherence

The intervention lasted 30 consecutive days. Participants received sufficient product for the full period and returned containers at the end of the study. Detailed adherence-assessment procedures are provided in Supplementary Methods S4.

### Outcome Variables

Primary variable: Change in sleep quality measured using the SQQ, a validated questionnaire completed daily by participants. The SQQ evaluates difficulty sleeping, daytime sleepiness, and total sleep quality score, with higher scores indicating poorer sleep quality.

Secondary variable: Change in phase angle measured hand-to-hand using the BIA-TELELAB device. Measurements were taken under standardised conditions (4-h fast, no intense exercise in the previous 24 h, empty bladder) at baseline (day 0) and at the end of the intervention (day 30).

Safety variables: Adverse events were monitored through open-ended questions at each participant contact and through a daily symptom diary.

Assessments were conducted at baseline and day 30, with daily SQQ completion throughout the intervention. The complete assessment schedule is provided in Supplementary Methods S5.

### Statistical Analysis

Analyses followed a modified intention-to-treat approach including all randomised participants with at least one post-baseline measurement. Missing outcome data were handled using linear mixed-effects models under a missing-at-random assumption, without imputation of missing values. Sleep variables were analysed using linear mixed models with repeated measures, including treatment group, time, and their interaction as fixed effects and participant as a random effect. Differences in longitudinal sleep outcomes between groups were assessed through the time × group interaction. Phase angle changes were compared between groups using the Mann-Whitney U test and within groups using the Wilcoxon test. Effect size was calculated using Cohen’s d, with 0.2, 0.5, and 0.8 representing small, medium, and large effects. Statistical significance was set at *p* < 0.05 (two-tailed). Analyses were conducted using R version 4.x and SPSS version 27.

## Results and Discussion

### Participant Characteristics

Between August and September 2025, 137 candidates were assessed for eligibility; 100 met inclusion criteria and were randomised: 50 to the VERUM group and 50 to the PLACEBO group. During the study, 23 participants dropped out or were excluded: 12 in the VERUM group (10 voluntary withdrawal, 2 loss to follow-up) and 11 in the PLACEBO group (9 voluntary withdrawal, 2 loss to follow-up).

For the BIA analysis, 77 participants who completed both measurements were included (38 VERUM, 39 PLACEBO). For sleep quality analysis, 61 participants provided at least one post-baseline SQQ measurement and were included in the mixed-model analysis (32 VERUM, 29 PLACEBO). Missing sleep data (16 participants; 6 VERUM, 10 PLACEBO) were primarily due to participant withdrawal and incomplete completion of scheduled daily questionnaires.

Baseline characteristics were similar between groups. Mean age was 47.8 ± 8.2 years (range 35–68), with 67.5% women (52/77) and 32.5% men (25/77). No significant baseline demographic differences were observed between groups (*p* > 0.05), confirming effective randomisation.

### Effects on Sleep Quality

**Fig. 1:**
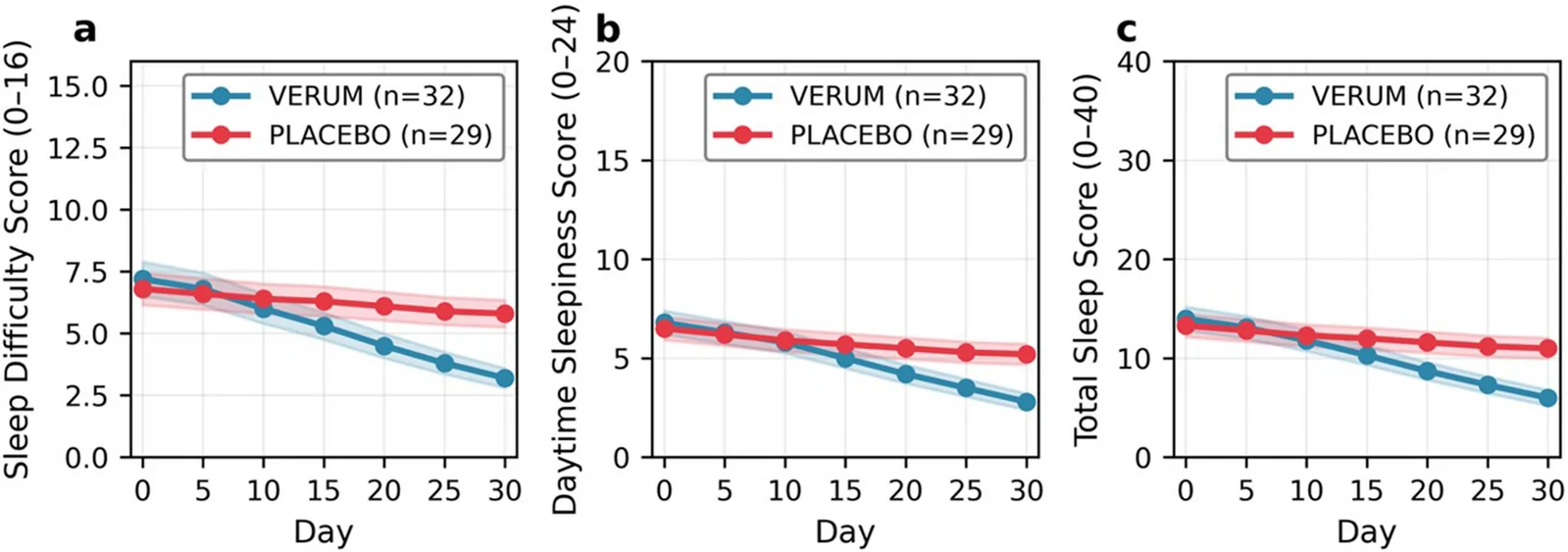
Changes in subjective sleep outcomes over the 30-day study period in the verum and placebo groups

Results in the VERUM group showed consistent and statistically significant improvements in subjective sleep outcomes (Fig. 1).

#### Difficulty sleeping

Scores decreased from 7.2 ± 2.1 to 3.2 ± 1.8 in the VERUM group (56% reduction, *p* < 0.001, d = 0.91), compared with 7.0 ± 2.3 to 5.5 ± 2.1 in the PLACEBO group (21% reduction, *p* = 0.08, d = 0.34). The time × group interaction was highly significant (*p* < 0.001) (Table 1).

**Table 1:** Changes in sleep quality: pre-post and between-group comparison

Variable	Group	PRE (x̄ ± SD)	POST (x̄ ± SD)	Change (x̄ ± SD)	Change (%)	*p*-value†	Cohen’s d	*p*-value
Difficulty of sleep	**VERUM (n = 32)**	7.2 ± 2.1	3.2 ± 1.8	-4.0 ± 2.3	-56	< 0.001	0.91	< 0.001
	**PLACEBO** **(n = 29)**	7.0 ± 2.3	5.5 ± 2.1	-1.5 ± 1.8	-21	0.08	0.34	
Daytime sleepiness	**VERUM (n = 32)**	6.8 ± 2.0	2.7 ± 1.6	-4.1 ± 2.1	-60	< 0.001	0.85	< 0.001
	**PLACEBO** **(n = 29)**	6.5 ± 2.2	5.2 ± 2.0	-1.3 ± 1.7	-20	0.12	0.31	
Total score	**VERUM (n = 32)**	14.0 ± 3.8	5.9 ± 3.2	-8.1 ± 4.1	-58	< 0.001	0.96	< 0.001
	**PLACEBO** **(n = 29)**	13.5 ± 4.1	10.7 ± 3.9	-2.8 ± 3.2	-21	0.09	0.33	

x̄ = sample mean. SD = standard deviation. †Wilcoxon test for intra-group comparisons. ‡p-value for time × group inter. action (longitudinal mixed models)

#### Daytime sleepiness

Scores decreased from 6.8 ± 2.0 to 2.7 ± 1.6 in the VERUM group (60% reduction, *p* < 0.001, d = 0.85), *versus* 6.5 ± 2.2 to 5.2 ± 2.0 in the PLACEBO group (20% reduction, *p* = 0.12, d = 0.31). The time × group interaction was highly significant (*p* < 0.001) (Table 1).

#### Total sleep quality score

The VERUM group decreased from 14.0 ± 3.8 to 5.9 ± 3.2 (58% reduction, *p* < 0.001, d = 0.96), while the PLACEBO group decreased from 13.5 ± 4.1 to 10.7 ± 3.9 (21% reduction, *p* = 0.09, d = 0.33). The time × group interaction was highly significant (*p* < 0.001) (Table 1).

**Fig. 2:**
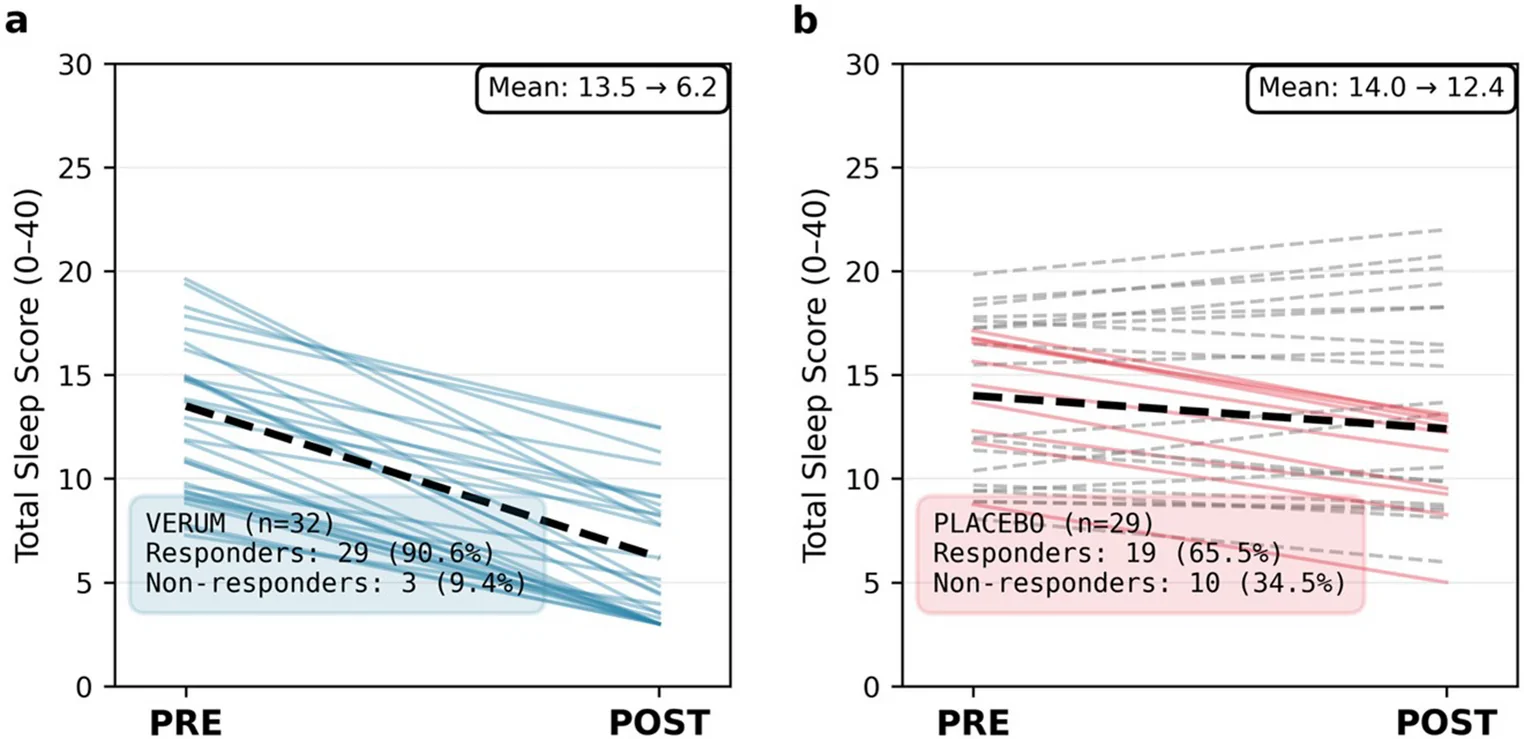
Individual changes in total sleep scores from baseline to post-intervention in the verum (**a**) and placebo (**b**) groups

Temporal analysis showed improvements in the VERUM group from the first week, remaining stable throughout the intervention (Fig. 1). Overall improvement in total sleep quality score was approximately 3.7 times greater in the VERUM group than in the PLACEBO group. Individual analysis (Fig. 2) showed improvement in 90.6% of VERUM participants (29/32) versus 65.5% in the PLACEBO group (19/29). Although improvements were greater in the VERUM group, a moderate improvement was also observed in the PLACEBO group, which should be considered when interpreting the magnitude of the intervention effect.

These results reflect perceived sleep quality and daytime functioning as assessed by validated self-report measures, rather than objective physiological sleep parameters.

### Effects on Phase Angle

Phase angle changes were minimal in both groups (Table 2). In the VERUM group, values decreased from 4.6 ± 0.8° to 4.4 ± 0.8° (mean change − 0.17 ± 0.33°, 3.8% reduction, *p* = 0.004). In the PLACEBO group, values decreased from 4.6 ± 0.7° to 4.5 ± 0.7° (mean change − 0.07 ± 0.26°, 1.6% reduction, *p* = 0.037). The time × group interaction was not significant (*p* = 0.32). Although both groups showed small statistically significant intra-group reductions, effect sizes were very small (d < 0.3) and likely not clinically relevant. Given the absence of between-group differences and small effect sizes, these changes are not considered clinically meaningful and are reported as exploratory outcomes only. No correlation was observed between changes in phase angle and improvements in sleep quality (*r*=-0.08, *p* = 0.52).

**Table 2:** Changes in body composition: pre-post and between-group comparison (*n* = 77)

Variable	Group	PRE (x̄ ± SD)	POST (x̄ ± SD)	Change (x̄ ± SD)	Change (%)	*p*-value†	*p*-value‡
Phase angle (°)	**VERUM (n = 38)**	4.6 ± 0.8	4.4 ± 0.8	-0.17 ± 0.33	-3.8	0.004	0.32
	**PLACEBO** **(n = 39)**	4.6 ± 0.7	4.5 ± 0.7	-0.07 ± 0.26	-1.6	0.037	

x̄ = sample mean. SD = standard deviation. †Wilcoxon test for intra-group comparisons. ‡Mann-Whitney U test for inter-group comparisons

### Adherence and Safety

Adherence to the protocol was excellent in both groups. No serious adverse events occurred, and no participants withdrew due to adverse events.

### Comparison with Previous Studies

The findings of this study suggest that the combination of vegetable protein (27 g/day) with ashwagandha and rhodiola is associated with greater improvements in perceived sleep quality compared to placebo over 30 days of intervention. The 56–60% reductions in sleep difficulty and daytime sleepiness, with large effect sizes (d = 0.85–0.96), represent improvements that suggest potential relevance for perceived sleep quality and daily functioning, although clinical significance should be interpreted cautiously given the subjective nature of the outcome and the improvement observed in the placebo group. The high proportion of participants reporting improvement in the VERUM group should be interpreted in the context of self-reported outcomes and potential expectancy effects. Our results are consistent with, but more pronounced than, those reported in previous studies on ashwagandha and sleep. Several recent clinical trials have documented that ashwagandha improves sleep quality with moderate to large effect sizes [10–12, 20, 21]. For example, a meta-analysis of five clinical trials found that ashwagandha significantly improved sleep quality (standardised mean difference: -0.59) and reduced anxiety in adults with insomnia [12]. However, most of these studies used ashwagandha as monotherapy, whereas our study evaluated a synergistic combination.

Rhodiola, although less specifically studied for sleep, has shown beneficial effects on fatigue and cognitive performance under stressful conditions [13, 14]. It is plausible that rhodiola contributes to the observed improvements in daytime sleepiness by optimising the adaptive response to stress and improving the quality of night-time rest.

Protein supplementation, on the other hand, has shown mixed results in previous studies. Some trials have reported improvements in sleep quality with tryptophan-rich proteins [22], while others have found no significant effects [23]. Our results suggest that the combination of plant protein with adaptogens may be more effective than either component alone, possibly due to synergistic mechanisms: proteins provide the substrates for the synthesis of sleep neurotransmitters (tryptophan → serotonin → melatonin), while adaptogens optimise the stress response and modulate the HPA axis, creating a more favourable neuroendocrine environment for sleep [24, 25].

Beyond sleep-related outcomes, recent data suggest that plant-based dietary patterns do not necessarily correspond to improvements in bioelectrical markers of cellular health. In a large study of young adult women, adherence to a vegetarian diet was independently associated with lower phase angle values and a slightly higher Extracellular Water to Total Body Water ratio, even after adjusting for BMI, smoking status, and physical activity [26]. Although the populations and study designs differ from ours, these findings are relevant because they indicate that plant-based eating patterns may be associated with distinct cellular bioelectrical profiles. The mechanisms underlying the observed improvements remain uncertain and were not directly assessed in this study. The following considerations are therefore hypothetical and should be interpreted as exploratory. Previous literature suggests that ashwagandha may influence the HPA axis, and has been shown to reduce cortisol levels and modulate the stress response [10, 11]. Chronic stress and hyperactivation of the HPA axis are well-established factors in the pathophysiology of insomnia [27]. It has been proposed that modulation of the stress response could contribute to improved sleep; however, this mechanism was not evaluated in the present study. In the case of GABAergic effects, withanolides, the active components of ashwagandha, have shown affinity for GABA-A receptors in preclinical studies [28]. The GABAergic system is the brain''''s main inhibitory system and plays a crucial role in inducing and maintaining sleep. This represents a potential explanatory pathway, although it cannot be confirmed based on the current data.

Regarding neurotransmitter optimization, plant protein provides tryptophan, a precursor to serotonin and melatonin. Although the relationship between dietary tryptophan and brain serotonin synthesis is complex (it depends on competition with other amino acids for transport across the blood-brain barrier), some studies have shown that tryptophan supplementation can improve sleep [27]. Combining it with adaptogens could optimise this process by reducing stress and inflammation, factors that can interfere with tryptophan metabolism. Although biologically plausible, the relationship between dietary tryptophan intake and sleep outcomes in this context was not directly measured. Both ashwagandha and rhodiola have anti-inflammatory and antioxidant properties [29, 30]. Low-grade chronic inflammation has been associated with sleep disorders, and the reduction of inflammatory markers could contribute to the improvements observed. However, inflammatory markers were not assessed in this study.

Unlike the consistent improvements in sleep quality, we did not observe significant changes in phase angle between groups. Both groups showed small statistically significant intra-group reductions, but these were very small in magnitude and probably not clinically relevant. There are several possible explanations for this lack of effect. First, the duration of the intervention could be insufficient. The phase angle reflects the integrity of cell membranes and body cell mass, parameters that probably require more than 30 days to show significant changes in response to nutritional interventions. Previous studies that have reported improvements in phase angle with protein supplementation have generally used longer intervention periods (12-24 weeks) and in populations with malnutrition or sarcopenia [17]. In addition, characteristics of the study population may have influenced these findings. Our participants were relatively healthy middle-aged adults reporting subjective sleep complaints without diagnosed sleep disorders. The phase angle may be more sensitive to nutritional interventions in populations with nutritional compromise or chronic disease, where there is greater room for improvement. Regarding the Protein dose, although 27 g/day is a reasonable dose, it may not be sufficient to induce measurable changes in body composition or cell mass in healthy adults over a short period.

### Limitations and Future Research

Methodological considerations of the hand-to-hand BIA method should be considered. Although the BIA-TELELAB device is convenient and non-invasive, hand-to-hand bioimpedance may be less sensitive than whole-body bioimpedance for detecting subtle changes in body composition. Furthermore, the 30-day intervention period is relatively short. We do not know whether the benefits are maintained in the long term or whether they could increase with longer interventions. Nor did we evaluate what happens when the supplement is discontinued. Moreover, although the SQQ questionnaire is a validated tool, it is based on the subjective perception of participants. We did not use objective methods such as polysomnography or actigraphy, which could provide more detailed information on sleep architecture and objective parameters (sleep latency, sleep efficiency, night-time awakenings, etc.). Therefore, findings cannot be interpreted as direct evidence of changes in sleep architecture or physiological sleep parameters. No formal correction for multiple comparisons was applied due to the exploratory nature of secondary and subgroup analyses; this increases the risk of type I error and is acknowledged as a limitation. In addition, our participants were relatively healthy adults with subjective complaints of poor sleep quality but no formal diagnosis of sleep disorder. The results may not be generalisable to patients with severe chronic insomnia, sleep apnoea, or other diagnosed sleep disorders. Although the combination of vegetable protein with ashwagandha and rhodiola was effective, our design does not allow us to determine the relative contribution of each component. We do not know whether all three components are necessary or whether any of them are particularly important. Therefore, causal attribution to specific ingredients is not possible. Future studies with factorial designs could address this question. Given the attrition rate and outcome-specific missing data, estimates of effect magnitude should be interpreted with caution, particularly for subjective sleep outcomes. Finally, we did not measure sleep-related biomarkers (melatonin, cortisol, inflammatory cytokines) or neurotransmitters, which would have provided valuable information on the underlying mechanisms. The improvement observed in the placebo group further highlights the potential contribution of non-specific effects.

## Conclusions

This randomised, double-blind, placebo-controlled clinical trial suggests that the combination of vegetable protein (27 g/day) with ashwagandha and rhodiola may improve perceived sleep quality in adults with subjective sleep complaints. These findings support the potential of this combination as a well-tolerated nutritional approach for improving perceived sleep quality, although further studies incorporating objective sleep measures and longer follow-up are required. The intervention could be particularly valuable for people who prefer natural alternatives to conventional hypnotic medications.

## Supplementary Information

Below is the link to the electronic supplementary material.

Supplementary Material 1 (DOCX 26.0 KB)

## Data Availability

All data supporting the findings of this study are available within the paper and its Supplementary Information.
