# Supplementary material for: Effects on Sleep Quality and Bioelectrical Parameters of Cellular Health After Administration of a Plant Protein Supplement Combined with Ashwagandha and Rhodiola: A Randomized Controlled Trial
Source: Plant Foods Hum Nutr. 2026 Nov 11;81(4):136. doi: 10.1007/s11130-026-01585-y (PMC13754943; doi:10.1007/s11130-026-01585-y)
Supplement: Supplementary file 1 [file 11130_2026_1585_MOESM1_ESM.docx]

**TITLE:** Effects on Sleep Quality and Bioelectrical Parameters of Cellular Health After Administration of a Plant Protein Supplement Combined with Ashwagandha and Rhodiola: a randomized controlled trial

**Supplementary material - Methods S1. Eligibility criteria**

Adults aged 35–70 years with subjective complaints of poor sleep quality, but without a diagnosed sleep disorder requiring pharmacological treatment, were recruited. Inclusion criteria were: age 35–70 years, BMI 18.5–34.9 kg/m², baseline Sleep Quality Questionnaire (SQQ) [21] score ≥4 (indicating sleep problems), and willingness to maintain stable lifestyle habits during the study. Exclusion criteria included diagnosis of sleep apnoea or other sleep disorders requiring treatment; regular use of hypnotics, anxiolytics, or antidepressants; severe chronic diseases (advanced renal, hepatic, or cardiac failure, active cancer); pregnancy or breastfeeding; allergy to any supplement component; and consumption of supplements containing adaptogens or proteins within four weeks before study start.

**Supplementary material - Methods S2. Randomisation, allocation concealment and code management**

The allocation sequence was generated by an independent statistician not involved in participant recruitment, intervention delivery, outcome assessment, or statistical analysis of trial outcomes. Participant enrolment was performed by trained study investigators who had no access to the allocation sequence at any stage of recruitment. Group assignment was implemented by a third-party trial coordinator who opened sequentially numbered, opaque, sealed envelopes only after participant enrolment and baseline assessment had been completed.

Allocation concealment was ensured using sequentially numbered, opaque, tamper-evident envelopes prepared by an external contract organisation independent from the research team. The integrity of the concealment procedure was verified prior to trial initiation by an independent monitor.

The randomisation code was held exclusively by the external manufacturer and a third-party data safety coordinator and was not accessible to investigators until the database was locked and all statistical analyses were completed.

**Supplementary Methods S3. Botanical extract characterization and quality control**

Rhodiola rosea extract: Derived from the root of Rhodiola rosea L. The extract was produced using a hydroalcoholic extraction process with a drug extract ratio (DER) of 4–6:1 (native herb:extract). The extract was standardised to contain 3% rosavins and 1% salidroside (quantified by HPLC), according to the manufacturer’s certificate of analysis.

Withania somnifera extract (KSM-66®): Derived exclusively from the root of Withania somnifera L. Dunal, extracted using a “green chemistry” hydroalcoholic process with a DER of approximately 10–12:1. The extract was standardised to contain 5% withanolides, quantified by validated HPLC methods as reported in batch-specific quality documentation provided by the manufacturer.

For both botanical extracts, quality control included high-performance liquid chromatography (HPLC) fingerprinting to verify batch consistency. Analytical procedures followed validated methods with established selectivity, accuracy, and precision as reported by the manufacturer’s quality assurance documentation.

**Supplementary Methods S4. Compliance and adherence**

Compliance was assessed using a multi-method approach including (i) return-to-dispensing ratio based on container count, and (ii) daily self-reported intake logs completed by participants throughout the intervention period.

No biochemical markers (e.g., plasma metabolites) or digital adherence monitoring systems were employed. This represents a limitation of the present pragmatic nutritional intervention and is acknowledged in the Discussion.

**Supplementary Methods S5. Assessment procedures**

Baseline visit (day 0): Eligibility criteria were verified, informed consent obtained, demographic and anthropometric data (weight, height, BMI) recorded, and the first BIA measurement performed. Participants completed the first SQQ and received instructions on questionnaire completion and product intake.

Intervention period (days 1–29): Participants completed the SQQ daily via a digital platform. Weekly telephone contacts monitored adherence, resolved questions, and recorded adverse events.

Final visit (day 30): A second bioimpedance measurement was taken under the same conditions as baseline. Product containers were collected to verify adherence, the final SQQ questionnaire was completed, and a closing interview assessed adverse events and satisfaction.
